# Assessment of reporting quality of randomized controlled trials investigating the effects of inulin-type fructans supplementation on cardiovascular disease risk factors: A systematic survey

**DOI:** 10.1371/journal.pone.0292184

**Published:** 2024-01-02

**Authors:** Jhalok Ronjan Talukdar, Alexandro Chu, Anika Garg, Fariha Chowdhury, Hope E. Harnack, Louise Huang, Claudia Sikorski, Lawrence Mbuagbaw, Russell J. de Souza

**Affiliations:** 1 Department of Health Research Methods, Evidence, and Impact, McMaster University, Hamilton, Ontario, Canada; 2 Department of Medicine, McMaster University, Hamilton, Ontario, Canada; 3 Faculty of Health Sciences, McMaster University, Hamilton, Ontario, Canada; 4 School of Rehabilitation Sciences, McMaster University, Hamilton, Ontario, Canada; 5 Department of Kinesiology, McMaster University, Hamilton, Ontario, Canada; 6 Population Health Research Institute, Hamilton Health Sciences Corporation, Hamilton, Ontario, Canada; 7 Department of Anesthesia, McMaster University, Hamilton, Ontario, Canada; 8 Department of Pediatrics, McMaster University, Hamilton, Ontario, Canada; 9 Biostatistics Unit, Father Sean O’Sullivan Research Centre, St Joseph’s Healthcare, Hamilton, Ontario, Canada; 10 Centre for Development of Best Practices in Health (CDBPH), Yaoundé Central Hospital, Yaoundé, Cameroon; 11 Department of Global Health, Stellenbosch University, Cape Town, South Africa; 12 Toronto 3D Knowledge Synthesis and Clinical Trials Unit, Clinical Nutrition, St Michael’s Hospital, Toronto, ON, Canada; 13 Clinical Nutrition & Risk Factor Modification Centre, St. Michael’s Hospital, Toronto, Ontario, Canada; 14 Li Ka Shing Knowledge Institute, St. Michael’s Hospital, Toronto, Ontario, Canada; 15 Global Health Graduate Program, McMaster University, Hamilton, Ontario, Canada; Faculty of Medicine and Biomedical Sciences, the University of Yaoundé I, Yaoundé, Cameroon, CAMEROON

## Abstract

**Background:**

Transparent and detailed reporting of randomized controlled trials (RCTs) is essential to judge its validity and generalizability. We assessed the reporting quality of RCTs examining the effects of inulin-type fructans supplementation on cardiovascular risk factors, before and after the publication of the Consolidated Standards of Reporting Trials (CONSORT) in 2010.

**Methods:**

We searched MEDLINE, EMBASE, Emcare, AMED, the Cochrane Library, and CINAHL from inception to May 15, 2022, including the reference lists of selected RCTs. We screened titles and abstracts and extracted the data independently and in duplicate. We included RCTs that investigated the effects of inulin-type fructans on cardiovascular disease risk factors (e.g., low-density lipoprotein cholesterol, triglycerides, fasting blood glucose) in adults (18 years or older). The primary outcomes of this study were: the overall reporting quality of RCTs (defined as the total number of items [0 to 36] present from the CONSORT checklist) published before and after CONSORT; and the study characteristics (e.g., sample size, significance of primary outcome) predictive of the CONSORT score. The secondary outcome was the reporting of each specific item of the CONSORT checklist during pre- and post-CONSORT periods. The mean difference in the total number of reported items in studies published before and after CONSORT were compared using a t-test and Poisson regression to explore the factors associated with overall reporting quality of RCTs. We used Fisher’s exact test to compare the adherence to each of the 36 items during pre- and post-CONSORT periods.

**Results:**

We identified 1,767 citations from our systematic search, of which 55 were eligible. There was a significant increase in the reporting of CONSORT items (mean difference 8.5, 95% confidence interval [CI] 5.24 to 11.71) between studies published before and after publication of CONSORT. The sole variable that was predictive of better reporting quality of RCTs was whether the study was published before or after CONSORT (incidence rate ratio 1.67, 95% CI 1.40 to 2.02). Completeness of reporting of RCTs only improved in 15 out of 36 items (41.6%) after the publication of CONSORT.

**Conclusion:**

The completeness of reporting in RCTs investigating inulin-type fructans supplementation on cardiovascular disease risk factors remains inadequate after the publication of CONSORT. Greater adherence to CONSORT by authors and enforcement of CONSORT by journals may improve the quality of reporting among RCTs.

## Introduction

Randomized controlled trials (RCTs) are considered the gold standard for assessing the effectiveness of health interventions [1–3]. A well-conducted RCT can transform patient care. However, reporting of the study design, conduct, analysis, and interpretation of a published RCT must be transparent and sufficiently detailed, such that readers and practitioners can appropriately judge the validity and applicability of the trial to particular practice settings [4]. This may be difficult to do when the reporting of trials is inadequate [5]. At its extreme, inadequate reporting can lead to a biased estimate of the treatment effect, leading physicians to avoid effective treatments or promote ineffective treatments [6]. The Consolidated Standards of Reporting Trials (CONSORT) statement was created in 2010 to facilitate improved and transparent reporting of trials by study authors [7]. CONSORT is a 37-item checklist that authors are expected to adhere to while reporting an RCT. The checklist assesses quality of reporting in an RCT in five broad domains: title and abstract, introduction, methods, results, and discussion.

Previous studies found that inadequately reported RCTs led to an overestimation of intervention effects, compared to adequately reported RCTs [6, 8]. Moher and colleagues compared studies published in three journals that adopted CONSORT (The British Medical Journal, The Journal of the American Medical Association, and The Lancet) compared to one that did not (The New England Journal of Medicine), and found that the adoption of CONSORT led to an overall 10% improvement in reporting quality of RCTs [9]. However, previous publications that assessed reporting quality of RCTs using CONSORT in COVID-19 [10], otolaryngologic [11], chiropractic [12], and in otorhinolaryngologic research studies [13] found inadequate reporting quality.

There is limited evidence examining the quality of reporting in RCTs in regard to its design, analysis, and interpretation, especially in the field of nutrition. As a case example, we examined the reporting quality of published trials assessing the effects of inulin-type fructans supplementation on cardiovascular risk factors. This study is part of a two-study series that a) assessed the reporting quality of abstracts (see “S1 File”) and b) in full-text reports of RCTs. The objectives of this study were to assess: 1) the overall reporting quality of these published RCTs before and after the publication of the CONSORT; 2) the factors that predicted reporting quality; and 3) the frequency of reporting of each item in the CONSORT checklist. The overall reporting quality was defined as the total number of items reported out of 36 items in the CONSORT checklist. We excluded one item (item 17b) as it assesses the reporting of binary outcomes, whereas the included RCTs in our study only reported continuous outcomes.

## Materials and methods

### Study design

We conducted a systematic survey of the literature with a comparison of mean, and domain-specific CONSORT scores, before and after CONSORT publication [1]. We established the methods of the study *a priori* and published the study protocol [14].

### Study selection

A comprehensive search strategy was developed by a librarian (LB) (see “S1 File”) to search RCTs in MEDLINE, EMBASE, Emcare, AMED, the Cochrane Library, and CINAHL from database inception to May 15, 2022, without language restriction. We supplemented the database search with a reference search of included RCTs.

### Inclusion and exclusion criteria

We reported the selection criteria for this study in detail in the published protocol [14]. In brief, we included RCTs that investigated the effects of inulin-type fructans (ITF) on cardiovascular disease risk factors (i.e., low-density lipoprotein cholesterol, triglycerides, fasting blood glucose, body mass index, body weight, waist circumference, waist-to-hip ratio, systolic and diastolic blood pressure, high-density lipoprotein cholesterol, very-low-density lipoprotein cholesterol, total cholesterol, apolipoproteins A1 and B, and hemoglobin A1c) in adults (18 years or older). We excluded trials that only reported postprandial effects of ITF, that included participants with conditions that seriously altered normal digestion or absorption of nutrients (e.g., chemotherapy, dialysis, liver disease), or included participants undergoing treatments with the same effects. We also excluded trials that included pregnant or lactating participants.

Reviewers (from JRT, AG, FC, LH, AC, HH, CS) screened the titles and abstracts, and assessed the eligibility of full-texts independently and in duplicate. Disagreements were resolved through discussion or consultation with a third reviewer (RJdS).

### Data collection

We extracted study characteristics (e.g., name of journal, publication year, journal impact factor, funding source(s), CONSORT endorsement status by the journal, significance of the results of the primary outcome, sample size). We considered the first reported outcome as the primary outcome if the authors did not clearly define a primary outcome. Among the 37 items in the CONSORT checklist, we extracted data related to the adherence to 36 of the items, as one item (item 17b) assesses the reporting of binary outcomes, whereas the included RCTs in our study only reported continuous outcomes. We extracted data independently and in duplicate (from JRT, AG, FC, LH, AC, HH, CS) and resolved any disagreement through discussion or consultation with a third reviewer (RJdS).

### Outcomes

The primary outcomes of this study were 1) the overall reporting quality of RCTs based on adherence to the CONSORT checklist and 2) the factors associated with reporting quality. The overall reporting quality was defined as number of items reported out of 36 items of the CONSORT checklist. Study publication year (before vs. after the publication of CONSORT 2010), endorsement of the CONSORT statement by the journal (defined as the presence of specific instructions for the authors to follow the CONSORT statement or not during article submission), most recent journal impact factor (as a continuous variable), the statistical significance of primary outcome (significant vs. non-significant), funding status (industry-funded vs others) and sample size (≤ 100 vs > 100) were considered as predictors of reporting quality. The secondary outcomes were the reporting of each item of the CONSORT checklist.

### Statistical analyses

We reported study characteristics as means and standard deviations (SD) for continuous variables and count (percent) for categorical variables. Fisher’s exact test was used to compare the adherence of reporting each of the 36 items before and after publication of CONSORT statement. We reported unadjusted odds ratios (ORs) with 95% confidence interval (CI). We also used a t-test to compare the number of items reported before and after the publication of CONSORT, and report them as mean differences and 95% CIs. Poisson regression was used to explore the factors associated with overall reporting quality of RCTs. Following previous published literature [15–17], we adjusted the model for study publication year (before or after the publication of CONSORT), endorsement of the CONSORT statement by the journal (whether there are specific instructions for the authors to follow the CONSORT statement or not), journal impact factor (as a continuous variable), the statistical significance of the primary outcome (significant vs non-significant), funding status (industry-funded vs others) and sample size (≤ 100 vs > 100). Our primary analysis used the impact factor of each journal for 2021–22 as a covariate. A sensitivity analysis used journal impact factor values for the year of publication of each included study. We hypothesized that there was an association between these factors and overall reporting quality of RCTs. We reported results from the regression analysis as incidence rate ratios (IRR) with 95% CIs [18]. The IRR is obtained by exponentiating the Poisson regression coefficient [19], and is interpreted similarly to odds ratios, in which an IRR > 1 indicates that RCTs published after CONSORT are reported at a higher quality than those published before CONSORT. For example, IRR 1.21 indicates that reporting quality of RCTs published after CONSORT is 1.21 times better than the reporting quality RCTs published before CONSORT [20]. Statistical significance was set at α = 0.05. We used residual deviance to understand goodness-of-fit of the overall Poisson regression model. We compared the adjusted model with unadjusted model with Akaike information criterion (AIC). A lower value of AIC indicated better fit. We analyzed the data with R version 4.0.3 [21].

## Results

### Study characteristics

**Fig 1:**
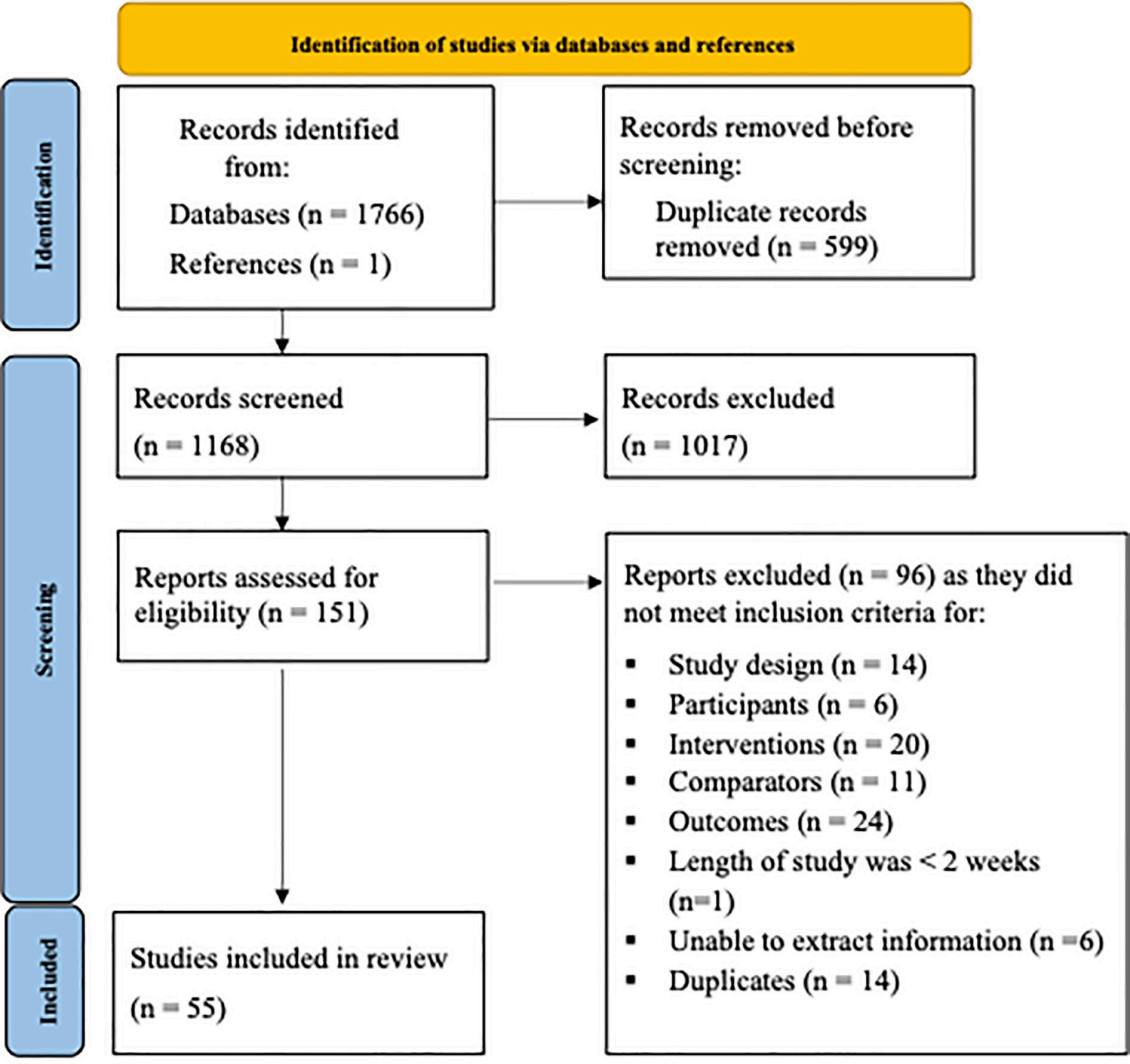
PRISMA flow diagram. Flow of studies through the systematic search, assessment of eligibility and inclusion into the review [28].

We identified 1,767 citations from the systematic search, of which 55 reports (see “S1 File”) met our eligibility criteria. Among these 55 articles, 13 were published before and 42 were published after the publication of CONSORT (**Fig 1**). Sixty-seven per cent of the studies reported statistically significant primary outcomes, 91% had a sample size less than or equal to 100 participants, 73% were published in journals that currently endorsed the CONSORT statement, and the mean impact factor of the publishing journal was 4.95 (SD 4.00) (**Table 1**).

**Table 1:** Characteristics of trials by period of publication.

	Pre-CONSORT Until 2009 (n = 13)	Post-CONSORT 2010–2022 (n = 42)	Total n = 55
Characteristics	n (%)	n (%)	n (%)
Statistically significant results			
Yes	7 (54)	30 (71)	37 (67)
No	6 (46)	12 (29)	18 (33)
Sample size			
≤100	13 (100)	37 (88)	50 (91)
>100	0 (0)	5 (12)	5 (9)
Funding			
Industry	5 (38.5)	9 (21.5)	14 (25.5)
Others	3 (23)	24 (57)	27 (49)
No information	5 (38.5)	9 (21.5)	14 (25.5)
*CONSORT endorsement by journals			
Yes	NA	40 (76)	40 (73)
No	NA	15 (24)	15 (27)
Journal’s impact factor (2021–2022) Mean (SD)	5.68 (1.95)	4.73 (4.49)	4.95 (4.0)
**Journal’s impact factor (time of publication)	2.89 (1.74)	3.65 (3.52)	3.45 (3.17)

CONSORT = Consolidated Standards of Reporting Trials; NA = not applicable

*CONSORT endorsement by journals until June 2022

**Available for n = 52 publications

### The overall reporting quality in randomized controlled trials

The mean number of items reported after the publication of CONSORT (mean 20.8, SD 5.38) was greater than before its publication (mean 12.3, SD 3.90); unadjusted mean difference (MD) of 8.5 (95% CI 5.24 to 11.71).

Study publication year (IRR 1.67, 95% CI 1.40 to 2.02) was significantly associated with reporting more items from the CONSORT checklist when adjusted for the endorsement of CONSORT by the journal, statistical significance of the results, sources of funding, sample size, and the journal’s impact factor. The unadjusted model also provided similar results (IRR 1.69, 95% CI 1.43 to 2.00). The unadjusted model (AIC: 337.54) had a better fit compared to the adjusted model (AIC: 345.64). **Table 2** presents the adjusted IRRs for the total number of reported CONSORT items.

**Table 2:** Adjusted incidence rate ratios for total number of CONSORT items.

	IRR (95% CI)	p-value
Intercept	11.22 (8.77 TO 14.29)	<0.001
CONSORT		
Pre (reference)		
Post	1.67 (1.40 to 2.02)	<0.001
*CONSORT endorsement by journals		
No (reference)		
Yes	1.09 (0.94 to 1.26)	0.257
Results statistically significant		
No (reference)		
Yes	0.99 (0.86 to 1.14)	0.864
Funding source		
Industry		
Other than industry	1.07 (0.91 to 1.27)	0.407
No information	0.96 (0.80 to 1.16)	0.658
Sample size		
Less than/equal to 100		
>100	1.09 (0.88 to 1.35)	0.409
Journal’s impact factor (2021–2022) (continuous)	1.00 (0.99 to 1.02)	0.748
**Journal’s impact factor at the time of publication (continuous)	1.01 (0.99 to 1.03)	0.242

IRR = Incidence rate ratio; CI = Confidence interval

**Note:** IRR < 1 indicates better reporting in reference group compared to the other group(s). It is the opposite for IRR>1. IRR = 1 indicates similar reporting in reference and other group (s).

*CONSORT endorsement by journals until June 2022

**Available for n = 52 publications

### Reporting of each item of the CONSORT checklist

We found statistically significant improvement in completeness of reporting of RCTs for 15 out of 36 items after the publication (2010 and later) compared to before publication (prior to 2010) of the CONSORT statement. After publication of the CONSORT statement, more studies could be identified as RCTs through the titles and abstracts (OR and 95% CI not estimable [NE], p <0.001), more RCTs reported methods for random sequence generation (OR 15.32, 95% CI 1.94 to 709.02, p = 0.003), type of randomization (OR 9.59, 95% CI 1.21 to 444.60, p = 0.019), allocation concealment (OR 9.59, 95% CI 1.21 to 444.60, p = 0.019), implementation of randomization (OR and 95% CI NE, p = 0.005), blinding (OR 11.57, 95% CI 1.47 to 535.36, p = 0.008), methods for additional analyses (e.g., subgroup, adjusted analyses) (OR and 95% CI NE, p <0.001), provided participant flow diagrams (OR 13.27, 95% 2.66 to 92.57, p = <0.001), recruitment information (OR and 95% CI NE, p = 0.011), baseline information (OR 15.82, 95% CI 2.28 to 190.78, p = 0.001), number analyzed (OR 4.27, 95% CI 1.15 to 15.95, p = 0.037), ancillary analyses (OR and 95% CI NE, p = <0.001), discussed limitations (OR 10.50, 95% CI 1.91 to 110.37, p = 0.002), registered protocols (OR 31.50 95% CI 3.89 to 1477.22, p = <0.001), and made the protocol publicly accessible (OR 22.69, 95% 2.85 to 1055.06, p = <0.001). The remaining items were reported similarly before and after publication of the CONSORT statement. Among the five domains of CONSORT (i.e., title and abstract, introduction, methods, results and discussion), after the publication of the CONSORT statement, the items composing the results domain were the most completely reported, followed by the methods domain. **Table 3** presents the frequency of reporting of each CONSORT item.

**Table 3:** Frequency of reporting each Consolidated Standards of Reporting Trials (CONSORT) item.

Section/Topic	Item No	Checklist item	Pre-CONSORT Until 2009 [N = 13] N (%)	Post-CONSORT 2010–2022 [N = 42] N (%)	Univariate analysis OR (95% CI)	P value
Title and abstract						
	1a	Identification as a randomised trial in the title	0 (0.00)	23 (54.76)	NE	<0.001
	1b	Structured summary of trial design, methods, results, and conclusions (for specific guidance see CONSORT for abstracts)	0 (0.00)	3 (7.14)	NE	>0.999
**Introduction**						
Background and objectives	2a	Scientific background and explanation of rationale	13 (100)	42 (100)	NE	NE
	2b	Specific objectives or hypotheses	13 (100)	42 (100)	NE	NE
**Methods**						
Trial design	3a	Description of trial design (such as parallel, factorial) including allocation ratio	10 (76.92)	18 (42.86)	0.23 (0.04 to 1.07)	0.055
	3b	Important changes to methods after trial commencement (such as eligibility criteria), with reasons	2 (15.38)	17 (40.48)	3.66 (0.67 to 38.15)	0.180
Participants	4a	Eligibility criteria for participants	12 (92.31)	40 (95.24)	1.65 (0.03 to 34.33)	0.562
	4b	Settings and locations where the data were collected	4 (30.77)	23 (54.76)	2.67 (0.62 to 13.82)	0.205
Interventions	5	The interventions for each group with sufficient details to allow replication, including how and when they were actually administered	13 (100)	42 (100)	NE	NE
Outcomes	6a	Completely defined pre-specified primary and secondary outcome measures, including how and when they were assessed	2 (15.38)	20 (47.62)	4.87 (0.90 to 50.52)	0.053
	6b	Any changes to trial outcomes after the trial commenced, with reasons	0 (0.00)	1 (2.38)	NE	>0.999
Sample size	7a	How sample size was determined	4 (30.77)	27 (64.29)	3.94 (0.91 to 20.63)	0.054
	7b	When applicable, explanation of any interim analyses and stopping guidelines	0 (0.00)	0 (0.00)	NE	NE
Randomisation:						
Sequence generation	8a	Method used to generate the random allocation sequence	1 (7.69)	24 (57.14)	15.32 (1.94 to 709.02)	0.003
	8b	Type of randomisation; details of any restriction (such as blocking and block size)	1 (7.69)	19 (45.24)	9.59 (1.21 to 444.60)	0.019
Allocation concealment mechanism	9	Mechanism used to implement the random allocation sequence (such as sequentially numbered containers), describing any steps taken to conceal the sequence until interventions were assigned	1 (7.69)	19 (45.24)	9.59 (1.21 to 444.60)	0.019
Implementation	10	Who generated the random allocation sequence, who enrolled participants, and who assigned participants to interventions	0 (0.00)	17 (40.48)	NE	0.005
Blinding	11a	If done, who was blinded after assignment to interventions (for example, participants, care providers, those assessing outcomes) and how	1 (7.69)	21 (50)	11.57 (1.47 to 535.36)	0.008
	11b	If relevant, description of the similarity of interventions	8 (61.54)	26 (61.90)	1.01 (0.22 to 4.28)	>0.999
Statistical methods	12a	Statistical methods used to compare groups for primary and secondary outcomes	13 (100)	42 (100)	NE	NE
	12b	Methods for additional analyses, such as subgroup analyses and adjusted analyses	0 (0.00)	23 (54.76)	NE	<0.001
**Results**						
Participant flow (a diagram is strongly recommended)	13a	For each group, the numbers of participants who were randomly assigned, received intended treatment, and were analysed for the primary outcome	3 (23.08)	34 (80.95)	13.27 (2.66 to 92.57)	<0.001
	13b	For each group, losses and exclusions after randomisation, together with reasons	7 (53.85)	34 (80.95)	3.54 (0.76 to 16.69)	0.071
Recruitment	14a	Dates defining the periods of recruitment and follow-up	0 (0.00)	16 (38.10)	NE	0.011
	14b	Why the trial ended or was stopped	0 (0.00)	0 (0.00)	NE	NE
Baseline data	15	A table showing baseline demographic and clinical characteristics for each group	7 (53.85)	40 (95.24)	15.82 (2.28 to 190.78)	0.001
Numbers analysed	16	For each group, number of participants (denominator) included in each analysis and whether the analysis was by original assigned groups	6 (46.15)	33 (78.57)	4.27 (1.15 to 15.95)	0.037
Outcomes and estimation	17a	For each primary and secondary outcome, results for each group, and the estimated effect size and its precision (such as 95% confidence interval)	13 (100)	42 (100)	NE	NE
	17b*	For binary outcomes, presentation of both absolute and relative effect sizes is recommended	NA	NA	NA	NA
Ancillary analyses	18	Results of any other analyses performed, including subgroup analyses and adjusted analyses, distinguishing pre-specified from exploratory	0 (0.00)	21 (50)	NE	<0.001
Harms	19	All important harms or unintended effects in each group (for specific guidance see CONSORT for harms)	10 (76.92)	25 (59.52)	0.45 (0.07 to 2.09)	0.333
**Discussion**						
Limitations	20	Trial limitations, addressing sources of potential bias, imprecision, and, if relevant, multiplicity of analyses	2 (15.38)	28 (66.67)	10.50 (1.91 to 110.37)	0.002
Generalisability	21	Generalisability (external validity, applicability) of the trial findings	0 (0.00)	8 (19.05)	NE	0.176
Interpretation	22	Interpretation consistent with results, balancing benefits and harms, and considering other relevant evidence	12 (92.31)	41 (97.62)	3.32 (0.04 to 273.91)	0.42
**Other information**						
Registration	23	Registration number and name of trial registry	1 (7.69)	31 (73.81)	31. 50 (3.89 to 1477.22)	<0.001
Protocol	24	Where the full trial protocol can be accessed, if available	1 (7.69)	28 (66.67)	22.69 (2.85 to 1055.06)	<0.001
Funding	25	Sources of funding and other support (such as supply of drugs), role of funders	0 (0.00)	3 (7.14)	NE	>0.999

NE = Not estimable, NA = Not applicable, *We excluded 17b as binary outcomes are not applicable for this study

## Discussion

### Main findings

This study provides a systematic assessment of the reporting quality of RCTs comparing studies published before and after the publication of the CONSORT statement. This study included RCTs that investigated the effects of ITF on CVD risk factors (e.g., low-density lipoprotein cholesterol, triglycerides, fasting blood glucose) in adults (18 years or older). We found statistically significant improvement in the completeness of reporting of RCTs for 15 out of 36 items when we compared studies published before and after the publication of CONSORT. After publication of CONSORT, more studies could be identified as RCT from titles and abstracts, reported methods for random sequence generation, allocation concealment, implementation of randomization, blinding, methods for additional analyses (e.g., subgroup, adjusted analyses), provided participant flow diagram, recruitment information, baseline information, number analyzed, ancillary analyses, discussed limitations, registered protocols, and made the protocol accessible to others. In adjusted models, publication of the CONSORT statement was significantly associated with improved quality of reporting, while other factors (e.g., significance of results, funding source, sample size) did not influence the quality of reporting.

Our findings suggests that there was significant improvement in the reporting of methodological details (e.g., specification of primary outcomes, random sequence generation, blinding) of RCTs after the publication of the CONSORT statement. Methodological details are important for assessing the quality of RCTs [22]. Especially, reporting approaches to sequence generation, blinding, allocation concealment, and handling of exclusions after allocation are the minimum requirement for assessing quality of RCTs [6]. Although there was an improvement in the reporting quality of certain CONSORT items after the publication of CONSORT, this improvement was inadequate. For example, despite an improvement in the reporting of the randomization methodology (e.g., sequence generation, allocation concealment, implementation of random allocation and blinding), these items were only reported in 40.48% to 61.90% of the studies. Inadequate reporting of the randomization methodology can lead to an overestimation of the treatment effect [8].

In addition, we did not find significant improvements in many of the CONSORT items. For example, there was no significant improvement in the reporting of abstract or sources of funding. This is particularly concerning because clinicians often make treatment decisions based on the abstracts of research articles [5, 23–25] due to time limitations, language barriers, or inaccessibility in accessing the full report [17]. Moreover, reporting a study’s sources of funding is necessary to determine potential risks of biases, as studies funded by pharmaceutical companies are often found to report more favourable results compared to studies funded by other sources [1]. Considering the impacts of the complete reporting of the CONSORT items, study authors, reviewers, and journals editors should be encouraged to collaborate to improve the reporting quality of RCTs.

### Relation to previous work

This is the first study to assess the reporting quality of RCTs that assessed the effects of ITF supplementation on CVD risk factors in adults. Consistent with our findings, studies published in other fields similarly reported suboptimal reporting quality of RCTs. For example, a recent study by Yin et al. [10] assessing reporting quality of RCTs in patients with COVID-19 found that the reporting rate was 53.85% based on the CONSORT checklist with all 37 items. Our study also found similar rate of reporting (on average 15 out of 36 or 58%). Huang et al. [11] assessed the reporting quality of RCTs in otolaryngology and reported a mean of 59% CONSORT adherence. Camm et al. [26] assessed reporting quality of RCTs relating to anti-arrhythmic agents and reported a mean score of 62% (15.4 of 25 group of items). A study by Nojomi et al. [27] assessed reporting quality of RCTs in Iranian journals and reported even lower CONSORT adherence (a mean of 43.8%).

Though our study reported similar increasing trends in the reporting quality of RCTs after the publication of the CONSORT statement, the absolute number of items reported remains concerningly low (41.6%). Observing the specific items of the CONSORT checklist, most of the items were not reported by the any of the RCTs even after publication of the CONOSRT statement.

### Limitations of the study

This study has inherent limitations. This study required making subjective judgements to score the items as reported or not. To minimize this limitation, we provided detailed instructions to the data extractors and conducted piloting and calibration exercises. The quality of reporting of RCTs could be influenced by various factors other than adherence to CONSORT that were not consider. For example, following a mandatory structure suggested by the journals (e.g., word count) or specific requirements by the funding agencies can lead to incomplete reporting. We followed published literature [15, 17] to adjust the factors that might influence incomplete reporting of RCTs. Another limitation of our study is that we defined CONSORT endorsement as whether the journal had endorsed CONSORT as at until June 2022. We made this decision considering the lack of information regarding specific time for CONSORT endorsement.

### Implications

Our study presents a snapshot of the current practice of reporting quality of RCTs investigating inulin-type fructans supplementation, which will highlight the items that are underreported. All the items of the CONSORT statement are important to assess the validity of an RCT. This finding might help the reviewers and journal editors to identify the gaps in certain domains and encourage the authors to make improvements in the reporting of those specific domain. A collaborative approach by authors, reviewers, and journal editors might lead to a greater number of reported CONSORT items in published RCTs.

## Conclusion

The completeness of reporting in RCTs investigating inulin-type fructans supplementation on cardiovascular disease risk factors was inadequate after the publication of CONSORT. Publication of the CONSORT statement had little or no impact on improving the reporting quality of RCTs. Authors are encouraged to adhere to the CONSORT statement and journals would benefit from further enforcing its usage.

## Supporting information

S1 Checklist The PRISMA 2020 statement includes a checklist of 27 items that cover the introduction, methods, results, and discussion sections of a report on a systematic review. (DOCX)

S1 File This file contains search strategy and reference list of included randomized control trials. (DOCX)

## Abbreviations

AIC: Akaike information criterion
CONSORT: Consolidated Standards of Reporting Trials
CVD: Cardiovascular disease
IRR: Incidence rate ratio
ITF: = Inulin-type fructans
MD: Mean difference
RCT: Randomized controlled trial
SD: Standard deviation
