# Supplementary material for: Assessment of reporting quality of randomized controlled trials investigating the effects of inulin-type fructans supplementation on cardiovascular disease risk factors: A systematic survey
Source: PLoS One. 2024 Jan 2;19(1):e0292184. doi: 10.1371/journal.pone.0292184 (PMC10760872; doi:10.1371/journal.pone.0292184)
Supplement: S1 File — (DOCX) [file pone.0292184.s002.docx]

**Supplementary materials**

Assessment of the quality of reporting in abstracts of randomized controlled trials investigating the effects of inulin-type fructans supplementation on cardiovascular disease risk factors: a systematic survey

***Search strategies***

**Database: OVID Medline Epub Ahead of Print, In-Process & Other Non-Indexed Citations, Ovid MEDLINE(R) Daily and Ovid MEDLINE(R)**

Search Strategy:

--------------------------------------------------------------------------------

1 (fructooligosaccharide* or fructo oligosaccharide*).ti,ab,kf.

2 neosugar*.ti,ab,kf.

3 Fructans/

4 Inulin/

5 (fructan* or inutest* or oligofructan* or polyfructosan*).ti,ab,kf.

6 inulin*.ti,ab,kf.

7 asteraceae.ti,ab,kf.

8 oligofructose*.ti,ab,kf.

9 chicory.ti,ab,kf.

10 chicory root.ti,ab,kf.

11 Helianthus/

12 jerusalem artichoke*.ti,ab,kf.

13 or/1-12

14 Lipids/

15 lipids.ti,ab,kf.

16 Lipoproteins/

17 Lipoproteins, IDL/

18 exp Lipoproteins, LDL/

19 exp Lipoproteins, HDL/

20 exp Lipoproteins, VLDL/

21 lipid.ti,ab,kf.

22 lipoprotein*.ti,ab,kf.

23 exp Triglycerides/

24 triglyceride*.ti,ab,kf.

25 triacetin/ or triolein/

26 triacylglycerol*.ti,ab,kf.

27 (HDL or LDL or VLDL or IDL or TG or TAG).ti,ab,kf.

28 cholesterol*.ti,ab,kf.

29 apolipoproteins a/ or apolipoprotein a-i/

30 apo* a1.ti,ab,kf.

31 apo* a i.ti,ab,kf.

32 (proapoliprotein adj1 (ai or a1 or a i or a-1)).ti,ab,kf.

33 Apolipoproteins B/

34 apo* b.ti,ab,kf.

35 or/14-34

36 exp waist circumference/

37 (waist adj3 (circumference* or ratio*)).ti,ab,kf.

38 Glucose/

39 glucose.ti,ab,kf.

40 blood pressure*.ti,ab,kf.

41 body mass index/

42 (body mass ind* or BMI).ti,ab,kf.

43 or/36-42

44 random*.ti,ab,kf.

45 rct*.ti,ab,kf.

46 randomized controlled trial/

47 randomized controlled trial.pt.

48 random allocation/

49 ((singl* or doubl* or tripl* or trebl*) adj3 (blind* or mask*)).ti,ab,kf.

50 exp clinical trial/

51 controlled clinical trial/

52 clinical trial*.ti,ab,kf.

53 or/44-52

54 13 and (35 or 43) and 53

55 remove duplicates from 54

***Database: Embase***

Search Strategy:

--------------------------------------------------------------------------------

1 fructose oligosaccharide/

2 (fructooligosaccharide* or fructo oligosaccharide* or oligofructose* or neosugar*).ti,ab,kw.

3 fructan/

4 (fructan* or inutest* or oligofructan* or polyfructosan*).ti,ab,kw.

5 inulin/

6 inulin*.ti,ab,kw.

7 asteraceae.ti,ab,kw.

8 chicory/

9 chicory.ti,ab,kw.

10 jerusalem artichoke/

11 jerusalem artichoke*.ti,ab,kw.

12 helianthus.ti,ab,kw.

13 or/1-12

14 lipid/

15 (lipid or lipids).ti,ab,kw.

16 lipoprotein/

17 intermediate density lipoprotein/

18 low density lipoprotein/

19 high density lipoprotein/

20 very low density lipoprotein/

21 lipoprotein*.ti,ab,kw.

22 (HDL or LDL or VLDL or IDL or TG or TAG).ti,ab,kw.

23 exp triacylglycerol/

24 (triglyceride* or triacylglycerol* or triacetin* or triolein*).ti,ab,kw.

25 cholesterol/

26 cholesterol*.ti,ab,kw.

27 apolipoprotein/ or apolipoprotein a/ or apolipoprotein a1/ or apolipoprotein b/ or apolipoprotein b100/ or apolipoprotein b48/

28 (apo* a1 or apo* a 1 or apo* a i or apo* ai).ti,ab,kw.

29 (proapoliprotein adj1 (ai or a1 or a i or a-1)).ti,ab,kw.

30 apo* b*.ti,ab,kw.

31 (proapoliprotein adj1 b*).ti,ab,kw.

32 or/14-31

33 waist circumference/

34 (waist adj3 (circumference* or ratio*)).ti,ab,kw.

35 glucose/

36 glucose*.ti,ab,kw.

37 blood pressure/

38 blood pressure*.ti,ab,kw.

39 body mass/

40 (body mass ind* or BMI).ti,ab,kw.

41 or/33-40

42 random*.ti,ab,kw.

43 rct*.ti,ab,kw.

44 randomized controlled trial/

45 exp randomization/

46 ((singl* or doubl* or tripl* or trebl*) adj3 (blind* or mask*)).ti,ab,kw.

47 clinical trial/

48 clinical trial*.ti,ab,kw.

49 controlled clinical trial/

50 or/42-49

51 13 and 41 and 50

52 remove duplicates from 51

***Database: Ovid Emcare***

Search Strategy:

--------------------------------------------------------------------------------

1 fructose oligosaccharide/

2 (fructooligosaccharide* or fructo oligosaccharide* or oligofructose* or neosugar*).ti,ab,kw.

3 fructan/

4 (fructan* or inutest* or oligofructan* or polyfructosan*).ti,ab,kw.

5 inulin/

6 inulin*.ti,ab,kw.

7 asteraceae.ti,ab,kw.

8 chicory/

9 chicory.ti,ab,kw.

10 jerusalem artichoke/

11 jerusalem artichoke*.ti,ab,kw.

12 helianthus.ti,ab,kw.

13 or/1-12

14 lipid/

15 (lipid or lipids).ti,ab,kw.

16 lipoprotein/

17 intermediate density lipoprotein/

18 low density lipoprotein/

19 high density lipoprotein/

20 very low density lipoprotein/

21 lipoprotein*.ti,ab,kw.

22 (HDL or LDL or VLDL or IDL or TG or TAG).ti,ab,kw.

23 exp triacylglycerol/

24 (triglyceride* or triacylglycerol* or triacetin* or triolein*).ti,ab,kw.

25 cholesterol/

26 cholesterol*.ti,ab,kw.

27 apolipoprotein/ or apolipoprotein a/ or apolipoprotein a1/ or apolipoprotein b/ or apolipoprotein b100/ or apolipoprotein b48/

28 (apo* a1 or apo* a 1 or apo* a i or apo* ai).ti,ab,kw.

29 (proapoliprotein adj1 (ai or a1 or a i or a-1)).ti,ab,kw.

30 apo* b*.ti,ab,kw.

31 (proapoliprotein adj1 b*).ti,ab,kw.

32 or/14-31

33 waist circumference/

34 (waist adj3 (circumference* or ratio*)).ti,ab,kw.

35 glucose/

36 glucose*.ti,ab,kw.

37 blood pressure/

38 blood pressure*.ti,ab,kw.

39 body mass/

40 (body mass ind* or BMI).ti,ab,kw.

41 or/33-40

42 random*.ti,ab,kw.

43 rct*.ti,ab,kw.

44 randomized controlled trial/

45 exp randomization/

46 ((singl* or doubl* or tripl* or trebl*) adj3 (blind* or mask*)).ti,ab,kw.

47 clinical trial/

48 clinical trial*.ti,ab,kw.

49 controlled clinical trial/

50 or/42-49

51 13 and 41 and 50

52 remove duplicates from 51

***Database: EBM Reviews - Cochrane Database of Systematic Reviews***

Search Strategy:

--------------------------------------------------------------------------------

1 (fructooligosaccharide* or fructo oligosaccharide* or neosugar* or (fructan* or inutest* or oligofructan* or polyfructosan*) or inulin* or asteraceae or oligofructose* or chicory or chicory root or jerusalem artichoke*).mp. [mp=title, short title, abstract, full text, keywords, caption text]

2 (lipids or lipid or lipoprotein* or triglyceride* or triacylglycerol* or (HDL or LDL or VLDL or IDL or TG or TAG) or cholesterol* or apo* a1 or apo* a i or (proapoliprotein adj1 (ai or a1 or a i or a-1)) or apo* b or (waist adj3 (circumference* or ratio*)) or glucose or blood pressure* or (body mass ind* or BMI)).mp. [mp=title, short title, abstract, full text, keywords, caption text]

3 1 and 2

***Database: AMED (Allied and Complementary Medicine)***

Search Strategy:

--------------------------------------------------------------------------------

1 (fructooligosaccharide* or fructo oligosaccharide* or neosugar* or (fructan* or inutest* or oligofructan* or polyfructosan*) or inulin* or asteraceae or oligofructose* or chicory or chicory root or jerusalem artichoke*).mp.

2 Lipids/

3 lipoproteins/

4 triglycerides/

5 (lipids or lipid or lipoprotein* or triglyceride* or triacylglycerol* or (HDL or LDL or VLDL or IDL or TG or TAG) or cholesterol* or apo* a1 or apo* a i or (proapoliprotein adj1 (ai or a1 or a i or a-1)) or apo* b or (waist adj3 (circumference* or ratio*)) or glucose or blood pressure* or (body mass ind* or BMI)).mp. [mp=abstract, heading words, title]

6 or/2-5

7 1 and 6

***Database: CINAHL***

Search Strategy:

--------------------------------------------------------------------------------

S1 fructose oligosaccharide* or fructooligosaccharide* or fructo oligosaccharide* or oligofructose* or neosugar*

S2 fructan* or inutest* or oligofructan* or polyfructosan*

S3 inulin* or asteraceae

S4 chicory

S5 jerusalem artichoke*

S6 helianthus

S7 S1 OR S2 OR S3 OR S4 OR S5 OR S6

S8 (MH "Lipids")

S9 lipid or lipids

S10 MH "Lipoproteins"

S11 lipoprotein*

S12 MH "Lipoproteins, LDL+"

S13 MH "Lipoproteins, HDL+"

S14 HDL or LDL or VLDL or IDL or TG or TAG

S15 MH "Triglycerides"

S16 triacylglycerol* or triacylglyceride*

S17 MH "Cholesterol"

S18 cholesterol*

S19 MH "Apolipoproteins"

S20 apo* a1 or apo* a 1 or apo* a i or apo* ai

S21 proapolioprotein N1 (ai or a1 or a i or a 1)

S22 apo* b*

S23 proapolioprotein N1 b*

S24 S8 OR S9 OR S10 OR S11 OR S12 OR S13 OR S14 OR S15 OR S16 OR S17 OR S18 OR S19 OR S20 OR S21 OR S22 OR S23

S25 MH "Waist Circumference"

S26 waist N3 (circumference* or ratio*)

S27 MH "Glucose"

S28 glucose*

S29 MH "Blood Pressure"

S30 blood pressure*

S31 MH "Body Mass Index"

S32 body mass ind* or BMI

S33 S25 OR S26 OR S27 OR S28 OR S29 OR S30 OR S31 OR S32

S34 S7 AND S24 AND S33

S35 S7 AND S24 AND S33

**Supplementary materials: References of included studies**

1. Aliasgharzadeh A, Khalili M, Mirtaheri E, Pourghassem Gargari B, Tavakoli F, Abbasalizad Farhangi M, et al. A Combination of Prebiotic Inulin and Oligofructose Improve Some of Cardiovascular Disease Risk Factors in Women with Type 2 Diabetes: A Randomized Controlled Clinical Trial. Advanced pharmaceutical bulletin. 2015;5(4):507-14.

2. Alles MS, de Roos NM, Bakx JC, van de Lisdonk E, Zock PL, Hautvast GA. Consumption of fructooligosaccharides does not favorably affect blood glucose and serum lipid concentrations in patients with type 2 diabetes. The American journal of clinical nutrition. 1999;69(1):64-9.

3. Bahmani F, Tajadadi-Ebrahimi M, Kolahdooz F, Mazouchi M, Hadaegh H, Jamal AS, et al. The Consumption of Synbiotic Bread Containing Lactobacillus sporogenes and Inulin Affects Nitric Oxide and Malondialdehyde in Patients with Type 2 Diabetes Mellitus: Randomized, Double-Blind, Placebo-Controlled Trial. Journal of the American College of Nutrition. 2016;35(6):506-13.

4. Blædel T, Holm JB, Sundekilde UK, Schmedes MS, Hess AL, Lorenzen JK, et al. A randomised, controlled, crossover study of the effect of diet on angiopoietin-like protein 4 (ANGPTL4) through modification of the gut microbiome. Journal of nutritional science. 2016;5:e45.

5. Bonsu NK, Johnson SJIJD, Metab. Effects of inulin fibre supplementation on serum glucose and lipid concentration in patients with type 2 diabetes. 2012;21:80-6.

6. Buddington RK, Kapadia C, Neumer F, Theis S. Oligofructose Provides Laxation for Irregularity Associated with Low Fiber Intake. Nutrients. 2017;9(12).

7. Castro-Sanchez F, Ochoa-Acosta D, Valenzuela-Rubio N, Domínguez-Rodríguez M, Fierros-Valdez J, Vergara-Jiménez MJAJNM. Inulin effect on weight loss and associated parameters with the development of cardiovascular disease in obese dyslipidemic subjects. 2017;4(1):1044.

8. Causey JL, Feirtag JM, Gallaher DD, Tungland BC, Slavin JL. Effects of dietary inulin on serum lipids, blood glucose and the gastrointestinal environment in hypercholesterolemic men. Nutrition Research. 2000;20(2):191-201.

9. Chambers ES, Byrne CS, Morrison DJ, Murphy KG, Preston T, Tedford C, et al. Dietary supplementation with inulin-propionate ester or inulin improves insulin sensitivity in adults with overweight and obesity with distinct effects on the gut microbiota, plasma metabolome and systemic inflammatory responses: a randomised cross-over trial. Gut. 2019;68(8):1430-8.

10. Clarke ST, Green-Johnson JM, Brooks SP, Ramdath DD, Bercik P, Avila C, et al. β2-1 Fructan supplementation alters host immune responses in a manner consistent with increased exposure to microbial components: results from a double-blinded, randomised, cross-over study in healthy adults. The British journal of nutrition. 2016;115(10):1748-59.

11. Cronin BE, Allsopp PJ, Slevin MM, Magee PJ, Livingstone MB, Strain JJ, et al. Effects of supplementation with a calcium-rich marine-derived multi-mineral supplement and short-chain fructo-oligosaccharides on serum lipids in postmenopausal women. The British journal of nutrition. 2016;115(4):658-65.

12. Crovesy L, El-Bacha T, Rosado EL. Modulation of the gut microbiota by probiotics and symbiotics is associated with changes in serum metabolite profile related to a decrease in inflammation and overall benefits to metabolic health: a double-blind randomized controlled clinical trial in women with obesity. Food & function. 2021;12(5):2161-70.

13. Daud NM, Ismail NA, Thomas EL, Fitzpatrick JA, Bell JD, Swann JR, et al. The impact of oligofructose on stimulation of gut hormones, appetite regulation and adiposity. Obesity (Silver Spring, Md). 2014;22(6):1430-8.

14. Davidson MH, Maki KC, Synecki C, Torri SA, Drennan KBJNR. Effects of dietary inulin on serum lipids in men and women with hypercholesterolemia. 1998;18:503-17.

15. Dehghan P, Farhangi MA, Tavakoli F, Aliasgarzadeh A, Akbari AM. Impact of prebiotic supplementation on T-cell subsets and their related cytokines, anthropometric features and blood pressure in patients with type 2 diabetes mellitus: A randomized placebo-controlled Trial. Complementary therapies in medicine. 2016;24:96-102.

16. Dehghan P, Gargari BP, Jafar-Abadi MA, Aliasgharzadeh A. Inulin controls inflammation and metabolic endotoxemia in women with type 2 diabetes mellitus: a randomized-controlled clinical trial. International journal of food sciences and nutrition. 2014;65(1):117-23.

17. Dewulf EM, Cani PD, Claus SP, Fuentes S, Puylaert PG, Neyrinck AM, et al. Insight into the prebiotic concept: lessons from an exploratory, double blind intervention study with inulin-type fructans in obese women. Gut. 2013;62(8):1112-21.

18. Fernandes R, Beserra BT, Mocellin MC, Kuntz MG, da Rosa JS, de Miranda RC, et al. Effects of Prebiotic and Synbiotic Supplementation on Inflammatory Markers and Anthropometric Indices After Roux-en-Y Gastric Bypass: A Randomized, Triple-blind, Placebo-controlled Pilot Study. Journal of clinical gastroenterology. 2016;50(3):208-17.

19. Forcheron F, Beylot M. Long-term administration of inulin-type fructans has no significant lipid-lowering effect in normolipidemic humans. Metabolism: clinical and experimental. 2007;56(8):1093-8.

20. Genta S, Cabrera W, Habib N, Pons J, Carillo IM, Grau A, et al. Yacon syrup: beneficial effects on obesity and insulin resistance in humans. Clinical nutrition (Edinburgh, Scotland). 2009;28(2):182-7.

21. Ghavami A, Roshanravan N, Nattagh-Eshtivani E, Sharifi S, Arzhang P, Sani MA, et al. The effect of high performance inulin on appetite, energy in-take and anthropometric indices in patient with type 2 diabetes. 2019;21:121-6.

22. Giacco R, Clemente G, Luongo D, Lasorella G, Fiume I, Brouns F, et al. Effects of short-chain fructo-oligosaccharides on glucose and lipid metabolism in mild hypercholesterolaemic individuals. Clinical nutrition (Edinburgh, Scotland). 2004;23(3):331-40.

23. Gómez-Reyes E, Orea-Tejeda A, Castillo-Martínez L, Cassis-Nosthas L, Vargas-Vorácková FJE. Prebiotics consumption modifies diastolic blood pressure, but does not affect serum lipids concentration in volunteers with ischemic heart disease. 2010;128:139.

24. Guess ND, Dornhorst A, Oliver N, Bell JD, Thomas EL, Frost GS. A randomized controlled trial: the effect of inulin on weight management and ectopic fat in subjects with prediabetes. Nutrition & metabolism. 2015;12:36.

25. Guess ND, Dornhorst A, Oliver N, Frost GS. A Randomised Crossover Trial: The Effect of Inulin on Glucose Homeostasis in Subtypes of Prediabetes. Annals of nutrition & metabolism. 2016;68(1):26-34.

26. Hiel S, Gianfrancesco MA, Rodriguez J, Portheault D, Leyrolle Q, Bindels LB, et al. Link between gut microbiota and health outcomes in inulin -treated obese patients: Lessons from the Food4Gut multicenter randomized placebo-controlled trial. Clinical nutrition (Edinburgh, Scotland). 2020;39(12):3618-28.

27. Holscher HD, Doligale JL, Bauer LL, Gourineni V, Pelkman CL, Fahey GC, et al. Gastrointestinal tolerance and utilization of agave inulin by healthy adults. Food & function. 2014;5(6):1142-9.

28. Jackson KG, Taylor GR, Clohessy AM, Williams CM. The effect of the daily intake of inulin on fasting lipid, insulin and glucose concentrations in middle-aged men and women. The British journal of nutrition. 1999;82(1):23-30.

29. Letexier D, Diraison F, Beylot M. Addition of inulin to a moderately high-carbohydrate diet reduces hepatic lipogenesis and plasma triacylglycerol concentrations in humans. The American journal of clinical nutrition. 2003;77(3):559-64.

30. Luo J, Rizkalla SW, Alamowitch C, Boussairi A, Blayo A, Barry JL, et al. Chronic consumption of short-chain fructooligosaccharides by healthy subjects decreased basal hepatic glucose production but had no effect on insulin-stimulated glucose metabolism. The American journal of clinical nutrition. 1996;63(6):939-45.

31. Luo J, Van Yperselle M, Rizkalla SW, Rossi F, Bornet FR, Slama G. Chronic consumption of short-chain fructooligosaccharides does not affect basal hepatic glucose production or insulin resistance in type 2 diabetics. J Nutr. 2000;130(6):1572-7.

32. Machado AM, da Silva NBM, Chaves JBP, Alfenas RCG. Consumption of yacon flour improves body composition and intestinal function in overweight adults: A randomized, double-blind, placebo-controlled clinical trial. Clinical nutrition ESPEN. 2019;29:22-9.

33. Mitchell CM, Davy BM, Ponder MA, McMillan RP, Hughes MD, Hulver MW, et al. Prebiotic inulin supplementation and peripheral insulin sensitivity in adults at elevated risk for type 2 diabetes: A pilot randomized controlled trial. Nutrients. 2021;13(9):3235.

34. Nishimura M, Ohkawara T, Kanayama T, Kitagawa K, Nishimura H, Nishihira J. Effects of the extract from roasted chicory (Cichorium intybus L.) root containing inulin-type fructans on blood glucose, lipid metabolism, and fecal properties. Journal of traditional and complementary medicine. 2015;5(3):161-7.

35. Padilla-Camberos E, Barragán-Álvarez CP, Diaz-Martinez NE, Rathod V, Flores-Fernández JM. Effects of Agave fructans (Agave tequilana Weber var. azul) on Body Fat and Serum Lipids in Obesity. Plant foods for human nutrition (Dordrecht, Netherlands). 2018;73(1):34-9.

36. Parnell JA, Reimer RA. Weight loss during oligofructose supplementation is associated with decreased ghrelin and increased peptide YY in overweight and obese adults. The American journal of clinical nutrition. 2009;89(6):1751-9.

37. Pedersen A, Sandström B, Van Amelsvoort JM. The effect of ingestion of inulin on blood lipids and gastrointestinal symptoms in healthy females. The British journal of nutrition. 1997;78(2):215-22.

38. Pol K, de Graaf C, Meyer D, Mars M. The efficacy of daily snack replacement with oligofructose-enriched granola bars in overweight and obese adults: a 12-week randomised controlled trial. British Journal of Nutrition. 2018;119(9):1076-86.

39. Rajkumar H, Kumar M, Das N, Kumar SN, Challa HR, Nagpal R. Effect of Probiotic Lactobacillus salivarius UBL S22 and Prebiotic Fructo-oligosaccharide on Serum Lipids, Inflammatory Markers, Insulin Sensitivity, and Gut Bacteria in Healthy Young Volunteers: A Randomized Controlled Single-Blind Pilot Study. Journal of cardiovascular pharmacology and therapeutics. 2015;20(3):289-98.

40. Reimer RA, Willis HJ, Tunnicliffe JM, Park H, Madsen KL, Soto-Vaca A. Inulin-type fructans and whey protein both modulate appetite but only fructans alter gut microbiota in adults with overweight/obesity: A randomized controlled trial. Molecular nutrition & food research. 2017;61(11).

41. Roshanravan N, Mahdavi R, Alizadeh E, Ghavami A, Rahbar Saadat Y, Mesri Alamdari N, et al. The effects of sodium butyrate and inulin supplementation on angiotensin signaling pathway via promotion of Akkermansia muciniphila abundance in type 2 diabetes; A randomized, double-blind, placebo-controlled trial. J Cardiovasc Thorac Res. 2017;9(4):183-90.

42. Russo F, Clemente C, Linsalata M, Chiloiro M, Orlando A, Marconi E, et al. Effects of a diet with inulin-enriched pasta on gut peptides and gastric emptying rates in healthy young volunteers. European journal of nutrition. 2011;50(4):271-7.

43. Salmean YAJPIN. Using inulin fiber supplementation with MyPlate recommendations promotes greater weight loss in obese women. 2019;21:81-5.

44. Satoh H, Kudoh A, Hasegawa K, Hirai H, Watanabe T. Yacon supplementation reduces serum free fatty acids and tumor necrosis factor alpha concentrations in patients with type 2 diabetes. Diabetology International. 2014;5(3):165-74.

45. Scheid MM, Genaro PS, Moreno YM, Pastore GM. Freeze-dried powdered yacon: effects of FOS on serum glucose, lipids and intestinal transit in the elderly. European journal of nutrition. 2014;53(7):1457-64.

46. Shakeri H, Hadaegh H, Abedi F, Tajabadi-Ebrahimi M, Mazroii N, Ghandi Y, et al. Consumption of synbiotic bread decreases triacylglycerol and VLDL levels while increasing HDL levels in serum from patients with type-2 diabetes. Lipids. 2014;49(7):695-701.

47. Sørensen M, Johansen OE. Idiopathic reactive hypoglycaemia - prevalence and effect of fibre on glucose excursions. Scandinavian journal of clinical and laboratory investigation. 2010;70(6):385-91.

48. Tajadadi-Ebrahimi M, Bahmani F, Shakeri H, Hadaegh H, Hijijafari M, Abedi F, et al. Effects of daily consumption of synbiotic bread on insulin metabolism and serum high-sensitivity C-reactive protein among diabetic patients: a double-blind, randomized, controlled clinical trial. Annals of nutrition & metabolism. 2014;65(1):34-41.

49. Tovar AR, Caamaño Mdel C, Garcia-Padilla S, García OP, Duarte MA, Rosado JL. The inclusion of a partial meal replacement with or without inulin to a calorie restricted diet contributes to reach recommended intakes of micronutrients and decrease plasma triglycerides: a randomized clinical trial in obese Mexican women. Nutrition journal. 2012;11:44.

50. Tripkovic L, Muirhead NC, Hart KH, Frost GS, Lodge JK. The effects of a diet rich in inulin or wheat fibre on markers of cardiovascular disease in overweight male subjects. Journal of human nutrition and dietetics : the official journal of the British Dietetic Association. 2015;28(5):476-85.

51. Vaghef-Mehrabany E, Ranjbar F, Asghari-Jafarabadi M, Hosseinpour-Arjmand S, Ebrahimi-Mameghani M. Calorie restriction in combination with prebiotic supplementation in obese women with depression: effects on metabolic and clinical response. Nutritional neuroscience. 2021;24(5):339-53.

52. van Dokkum W, Wezendonk B, Srikumar TS, van den Heuvel EG. Effect of nondigestible oligosaccharides on large-bowel functions, blood lipid concentrations and glucose absorption in young healthy male subjects. European journal of clinical nutrition. 1999;53(1):1-7.

53. Williams CJ, Torquati L, Li Z, Lea RA, Croci I, Keating E, et al. Oligofructose-Enriched Inulin Intake, Gut Microbiome Characteristics, and the VO2Peak Response to High-Intensity Interval Training in Healthy Inactive Adults. Journal of Nutrition. 2022;152(3):680-9.

54. Wong JM, Kendall CW, de Souza R, Emam A, Marchie A, Vidgen E, et al. The effect on the blood lipid profile of soy foods combined with a prebiotic: a randomized controlled trial. Metabolism: clinical and experimental. 2010;59(9):1331-40.

55. Ziaei R, Shahshahan Z, Ghasemi-Tehrani H, Heidari Z, Ghiasvand R. Effects of inulin-type fructans with different degrees of polymerization on inflammation, oxidative stress and endothelial dysfunction in women with polycystic ovary syndrome: A randomized, double-blind, placebo-controlled trial. Clinical Endocrinology. 2022.
